# Expanded perlite immobilization enhances long-term survival and mineralization activity of microbial spores in cementitious environments

**DOI:** 10.1128/aem.02456-25

**Published:** 2026-01-16

**Authors:** Lu Jiang, Qi Wang, Sisi Hu, Xiangbi Zhao, Wenjing Wang, Yu Zhang, Yuanzhen Liu

**Affiliations:** 1 School of Civil and Hydraulic Engineering, Ningxia University 56693, Yinchuan, China; 2 School of Architecture, Ningxia University 56693, Yinchuan, China; 3 Faculty of Engineering, China University of Geosciences 505014 , Wuhan, China; 4 College of Civil Engineering, Taiyuan University of Technology 47846, Taiyuan, China; Colorado School of Mines, Golden, Colorado, USA

**Keywords:** MICP, long-term activity, expanded perlite, self-healing

## Abstract

Microbially induced carbonate precipitation (MICP) provides a sustainable approach for the autonomous repair of microcracks in concrete. However, its practical application is limited by the poor long-term survival of microorganisms in the highly alkaline environment of cement matrices. This study used expanded perlite as an immobilization carrier to systematically investigate the effects of pH, temperature, and aging on microbial spore survival. Under non-immobilized conditions, acclimatized spores showed optimal long-term activity at pH = 10 and 0°C. After 180 days, the spore survival rate reached 12.31%, and urease activity achieved 0.74 mmol/(L·min)—approximately twice and nine times higher, respectively, than those recorded at 30°C over the same period. Although environmental factors reduced microbial mineralization capacity under immobilized conditions, mineral precipitation stabilized at around 5.60 g, representing a 28-fold increase compared to non-immobilized results over the same duration. These findings confirm that the carrier effectively alleviates the adverse effects of high alkalinity and temperature variations. The expanded perlite-based immobilization strategy significantly extended microbial service life, improved remediation efficiency, enhanced engineering feasibility, and reduced long-term maintenance costs. This research offers critical technical support for the development of durable and high-efficiency self-healing concrete systems.

**IMPORTANCE:**

Microbially induced carbonate precipitation (MICP) has gained significant attention as a promising technology in architecture and civil engineering. However, the understanding of microbial long-term activity and mineralization capacity within cement-based materials remains limited. This study investigated the influence of environmental factors on microbial spore survival in such materials by monitoring key indicators, including microbial concentration, urease activity, and mineral precipitation. Furthermore, it identified specific environmental conditions that support prolonged microbial viability. The use of expanded perlite as a carrier material aimed to mitigate external environmental stresses on microorganisms, thereby extending their mineralization capability over extended periods. These findings provide a scientific basis for the rational design of microbially mediated self-healing concrete systems.

## INTRODUCTION

Concrete remains the predominant construction material in civil engineering (1, 2). However, its inherent low tensile strength and brittleness make it susceptible to microcrack formation under mechanical loads and environmental exposure (3, 4). These cracks facilitate the ingress of water, oxygen, carbon dioxide, chlorides, and other corrosive agents, which degrade the mechanical properties and durability of concrete structures (5). Over time, microcracks propagate into macrocracks, compromising structural integrity and load-bearing capacity. Research has demonstrated that microbially induced carbonate precipitation (MICP) exhibits high compatibility with concrete matrices and significant potential for enhancing the durability and structural resilience of cement-based materials. This technology offers a promising approach to extending the service life of concrete structures while reducing long-term maintenance costs (6, 7).

Research indicates that urease activity and microbial mineralization capacity vary significantly across bacterial species, directly influencing the crack self-healing efficiency (8, 9). In cement mortar applications, nonureolytic bacteria demonstrate more consistent repair performance compared to ureolytic strains, which exhibit greater variability (10). The microalga *Nannochloropsis oceanica IMET1*, along with its bacterial symbionts, has shown potential for carbon capture, utilization, and storage under high-pH and high-alkalinity conditions, concurrently producing biomass and CaCO_3_ (11). Another approach involves *Methylocystis* parvus OBBP, which utilizes calcium formate as a substrate; methane supplementation further enhances its mineralization capacity (12). Ureolytic bacteria accelerate mineralization rates approximately 10^14^-fold compared to abiotic reactions, maintaining high activity in alkaline concrete environments and generating substantial mineral precipitates. These properties make them the most widely adopted microorganisms in self-healing concrete applications (13). Experimental results demonstrate that ureolytic bacteria consistently enhance compressive strength across concrete ages (14). For instance, *Lysinibacillus boronitolerans*, isolated from concrete, exhibits high urease activity and grows to OD_600_ ≈2.0 within 24 h at pH 13 (15). *XR1 marine Bacillus* reduces brittleness associated with conventional MICP (16). *Bacillus subtilis* and *Bacillus megaterium* increase concrete strength by 22.5% and 14.3% (17), respectively, while *Bacillus pasteurii* at 10^5^ cells/mL improves compressive strength by 36.4% (18). Additionally, bacterial incorporation reduces water absorption by 28.27% and enhances chloride corrosion resistance (19). *Bacillus subtilis* achieves 88% crack healing in seawater within 1 day (20), and aerobic bacteria repair cracks averaging 376 mm width within 10 days while significantly reducing permeability (21). However, microbial viability in concrete remains challenging: *Bacillus pasteurii* viability declines by 91% after 1 day in cement slurry, with only 1% and 0.2% survival after 7 and 28 days, respectively (22). *Bacillus megaterium* persists for 28 days (23), and *Bacillus cohnii* spores survive for less than 4 months (24). The sharp decline in microbial viability is primarily attributed to the confined, desiccated post-hydration environment, pore refinement, and mechanical stresses during concrete mixing and hardening (25).

The pH, temperature, and moisture conditions at concrete crack sites exhibit significant variation depending on crack location and age. Existing studies demonstrate that the local microenvironment directly governs microbial activity and mineralization efficiency. While the high alkalinity of concrete generally inhibits microorganisms, certain bacteria exhibit remarkable adaptability. *Lysinibacillus sphaericus* demonstrates both urea tolerance and high-pH resistance, achieving optimal growth at pH = 12 with a cell concentration of 6.8 × 10³ CFU/mL. Under these conditions, it generates 6.4 times more CaCO_3_ precipitation than at unregulated pH levels, with mineral precipitation increasing proportionally to medium alkalinity (26, 27). *Bacillus pasteurii* maintains peak activity at pH = 10 while surviving at pH 13. Under highly alkaline conditions (pH > 11), high bacterial concentrations permit sustained urease production within 24 h (28, 29). *Bacillus licheniformis* grows across pH 7–12, with optimal mineral precipitation at pH = 8, establishing it as an efficient biomineralization agent across diverse environments (30, 31). Temperature critically influences microbial metabolism and CaCO_3_ precipitation kinetics, affecting crystal size, cementation quality, and deposition rates (32, 33). Temperature critically influences microbial metabolism and CaCO_3_ precipitation kinetics, affecting crystal size, cementation quality, and deposition rates. Low temperatures inhibit enzymatic activity, material transport, and cellular replication, whereas elevated temperatures enhance substrate solubility through increased thermal expansion. Microbial urease activity peaks between 30°C and 40°C (34, 35). Although *Bacillus pasteurii* exhibits 28-fold slower growth at 40°C compared to 30°C, its optical density after 7 days of incubation reaches 1.6 times the initial value (36). In microbial self-healing concrete, 30°C yields maximum crack repair efficiency: complete healing for cracks <0.25 mm and 77.8% recovery for 0.25–0.45 mm cracks. Performance declines at both higher and lower temperatures (21). Comparative studies of normal, water, and wet-dry curing conditions confirm that biological mineralization requires free water as a reaction medium, though systematic investigation of moisture gradient effects remains incomplete.

Comprehensive research has established MICP as a pivotal technology for improving the mechanical properties and durability of concrete. The crack-repair capability of microbial self-healing concrete fundamentally relies on microbial viability and induced mineralization activity. The duration of effective self-healing extends with prolonged microbial persistence in the matrix, while the repair efficiency correlates directly with the level of microbial mineralization activity. Microbial spores are recognized for their sustained viability and long-term persistence within concrete environments. To date, research has primarily focused on short-term crack self-healing performance, examining factors such as initial crack width and nutrient composition. However, the synergistic evolutionary mechanisms governing long-term microbial activity and mineralization in cementitious systems remain poorly understood. This study systematically investigates the long-term survival and mineralization capacity of domesticated microbial spores in simulated cementitious matrices under varying environmental conditions, including temperature and pH. To mitigate viability loss caused by the confined, desiccated, high-pH post-hydration environment, mechanical stresses during mixing and pore refinement, carrier-based immobilization will be employed (25, 37). The findings from this work will provide innovative and practical technical support for designing high-performance, durable, and engineered self-healing concrete systems.

## MATERIALS AND METHODS

### Experimental materials

#### Microbial strain

The bacterial strain used in this study was *Bacillus pasteurii*, a Gram-positive, ureolytic bacterium capable of urease production. Its alkaliphilic nature enables survival in alkaline environments, while its ability to form spores provides resistance under adverse conditions.

#### Reagents

##### The domestication of microorganisms

The purchased bacterial culture was prepared as a suspension by mixing the stock culture with glycerol in equal proportions and stored under low-temperature conditions. The domestication process employed in this experiment was a gradient domestication method, with the following specific operational steps:

The aforementioned bacterial suspension was inoculated at a 1% inoculum rate into sterile medium adjusted to pH 7.5. Incubate for 24 h at 30°C and 150 rpm in a constant-temperature shaking incubator. Select the bacterial culture exhibiting the highest OD_600_ value, adjust the medium pH, and proceed with subsequent cultivation. The selected bacterial strain was sequentially inoculated into sterile culture media with pH values of 8.5, 9.0, 9.5, and 10.0 for screening, until the strain exhibiting the highest OD_600_ value under pH = 10 conditions was obtained. To ensure the stability of microbial adaptation, each gradient-adapted colony underwent a 3-day stabilization period before proceeding to the next adaptation stage.

##### Preparation of microbial spores

Research indicates that spore development can be enhanced by adding the right quantity of Mn^2+^ (38–40). In order to make the spore culture medium, MnSO_4_·H_2_O, soybean protein hydrolysate, trypsin hydrolysate, urea, and NaCl were added, and the pH was brought to 10. The specifications for the specific spore culture medium are shown in Table 1 below.

**TABLE 1:** The sporulation medium (g/L)

NaCl	Urea	Peptones, soybean	Tryptone	MnSO_4_·H_2_O
5	20	5	15	0.01

The centrifuge tubes, pipette tips, culture media, etc., should all be sterilized and allowed to cool. In 250 mL conical flasks with 100 mL of culture media, inoculate the strain at a rate of 1% after the culture medium has cooled to 40°C. Cover the flasks with sterile, breathable sealing film. Lastly, set the conical flask to 30°C and 160 rpm for 24 h in a constant-temperature shaking incubator.

##### Simulated cement-based pore solution

Stir well to combine cement and water in a 1:3 ratio. Initially, stir once every 2 h. Filter the supernatant once it has settled for a full day. 10% of the filtered cement simulation solution should be mixed with 0.1 mol/L N-cyclohexyl-3-aminopropanesulfonic acid solution. Then, using HCl and NaCl solutions, respectively, bring the simulation solution’s pH down to 7, 8, 9, 10, 11, and 12 to create the simulated cement-based pore solution.

##### Selection of expanded perlite

Research indicates that expanded perlite with a particle size of 1.18–2.36 mm exhibits more uniform distribution within concrete and exerts a lesser impact on its mechanical properties (41). Consequently, this study employs expanded perlite of this particle size as the carrier for immobilizing microorganisms.

This study employs the saturated water absorption rate to characterize the porosity of expanded perlite. In accordance with standard GB/T 17431.2-2010 “Lightweight Aggregates and Test Methods—Part 2: Test Methods for Lightweight Aggregates,” the water absorption rate of expanded perlite was determined to be 395.8% after adsorption for 15 min under a negative pressure of 0.06 MPa (21, 41, 42).

Consequently, it was established that when using expanded perlite with a particle size of 1.18–2.36 mm as the carrier material, the ratio of expanded perlite to bacterial suspension should be 1:4 for immobilizing microorganisms under 0.06 MPa negative pressure adsorption for 15 min.

##### “Sugar coating” expanded perlite sugar coating layer, protective layer

This experiment employs a coating system (sugar coating layer + protective layer) applied to the surface of expanded perlite to shield microorganisms and nutrients, preventing their loss during mixing; reduce the water absorption rate of expanded perlite; prevent premature contact between microorganisms and nutrients; and ensure the microbial concentration aligns with the nutrient concentration. It enhances the strength of expanded perlite to withstand mechanical stresses during concrete mixing; increases its brittleness to fracture at the onset of cracking, thereby releasing microorganisms; and improves the bond strength between perlite and the matrix. The specific material ratios for the sugar-coating layer and protective layer are detailed in Tables 2 and 3 (43).

**TABLE 2:** Sugar coating layer wrapping material (g)

Perlite	Water	Urea	Ca(NO_3_)_2_	Peptones, soybean	MgO	KH_2_PO_4_	CH_3_COONa	Tryptone
100	165	30	82	0.5	218	82	4.4	1.5

**TABLE 3:** Protective layer wrapping material (g)

Perlite	Water	Na_2_SiO_3_	NaOH	Metakaolin	CH_5_SiO_3_Na	C_11_H_12_O_2_
100	35	100	13.2	84.1	4.2	4.2

### Experimental methods

#### Experiments on the long-term survival characteristics of microorganisms in cement simulation solutions

##### Determination of bacterial concentration

Research indicates that within the range of 10^5^ to 10^8^ CFU/mL, the concentration of *Bacillus pasteurii* correlates directly with the optical density (OD) measured at 600 nm using a visible spectrophotometer (44). Consequently, this experiment employs the turbidimetric method to indirectly determine bacterial suspension concentration by measuring its absorbance at a specific wavelength.

Bacterial sludge samples were obtained by centrifuging bacterial suspensions, with temperature and pH serving as influencing factors. To investigate pH’s effect on microbial activity, bacterial sludge was resuspended using sterile simulated cement solutions with pH values ranging from 7 to 12 as diluents. Once the resuspension’s OD_600_ value decreased to 1.5, the bacterial solution was placed in 20-mL centrifuge tubes. To assess bacterial concentration over 0–270 days, centrifuge tubes were sealed with membrane caps and placed in a constant-temperature incubator set at 30°C. To investigate temperature effects on microbial survival, a cement-simulated solution at pH = 10 served as the diluent. After adjusting the bacterial suspension to an OD_600_ value of 1.5, it was placed in incubators maintained at temperatures ranging from 0°C to 40°C. Microbial concentrations were measured at predetermined time points (45). To minimize error, three centrifuge tubes were measured per age stage, with all experiments conducted through three independent replicates. Data are presented as means, with significance tests performed using the method ANOVA (*P* < 0.05 defined as statistically significant).

##### Assay of urease activity

*Bacillus pasteurii* produces urease during metabolism. Under the action of urease, urea hydrolyzes to form NH₄^+^ and CO₃²⁻, thereby increasing the solution’s conductivity. Whiffin’s research (46) demonstrated that the extent of urea hydrolysis is directly proportional to the change in solution conductivity. Consequently, this experiment employs a conductivity meter to measure conductivity variations in bacterial cultures at different growth stages under various environmental conditions, thereby assessing urease activity. The specific testing procedure is as follows: mix 5 mL of the bacterial suspension with 45 mL of urea solution (0.56 mol/L) to ensure uniformity, resulting in a final urea concentration of 0.5 mol/L in the mixture. Insert the electrode rod into the mixture and measure the change in conductivity (mS/cm) over 5 min. Calculate the change in conductivity per unit time (mS/[cm·min]), multiply by the dilution factor of 10, then determine microbial urease activity using the following formula:

UE=Δσ×11.11

where *UE* is the urea decomposed in mM, and *Δσ* is the conductivity in ms/cm.

### Long-term induced mineralization experiments of microorganisms in cement simulation solutions

The bacterial suspension of the test sample is prepared and maintained in accordance with the previously specified experiment, and the mineral production is assessed after the sample is left for 0–270 days. Relevant experiments indicate that when urea and calcium nitrate serve as nutrients, the induced mineralization yields a greater quantity of minerals, demonstrating excellent crack repair efficacy while exerting minimal impact on concrete’s mechanical properties. Consequently, this study employs urea (0.5 mol/L) and calcium nitrate (0.5 mol/L) as nutrient solutions, incorporated in a 1:1 ratio. Following a 24-h incubation period, the microbial culture solution was centrifuged, reconstituted in distilled water, and its OD_600_ was adjusted to 1.5. To obtain mineral precipitates, equivalent amounts of nutrient solution were added, and the nutrient solution and microbial suspension mixture were incubated for 24 h in a shaking incubator set at a constant temperature of 30°C. Centrifugation was used to gather the precipitates, which were then dried in an oven set at 105°C and weighed.

### Long-term induced mineralization experiments of microorganisms in cement simulated solutions under fixed loading conditions

After uniformly misting the bacterial solution over the enlarged expanded perlite protective layer and the dried sugar-coated layer, put everything in a dry and sterile beaker. After adding the simulated solution, incubate for 0, 1, 3, 7, 14, 28, 90, and 180 days at a steady temperature. Use filter paper to remove any leftover simulated solution from the beaker at each test age. Then, put the beaker and inoculated expanded perlite in a laminar flow hood for 15 min to sterilize them with UV light. Place the infected expanded perlite in a constant-temperature incubator set at 30°C after crushing it, and adding 80 g of nutrient solution, and covering it with plastic wrap. Following a 24-h period, use the filter paper from the previous stage to remove any surplus nutrient solution. Next, lay the expanded perlite on the filter paper and place the beaker in an oven set to 105°C until the mass no longer changes. Finally, determine the quantity of precipitate M_L_ (equation 1). In the equation, m_0_ is the mass of the beaker; m_1_ is the mass of the filter paper; m is the mass of the expanded perlite and filter paper after drying.


(1) $$M_{L}=m-m_{0}-m_{1}-80.$$

### Experiment on the effect of moisture content in cement simulated solution on long-term induced mineralization of microorganisms in a solid state

The moisture content of expanded perlite when it reaches saturation is 100%. Other moisture contents are expressed as a percentage of the saturated moisture content. To ensure consistency in the initial addition of bacterial solution, the 0% moisture content group collected 81.73 g of bacterial solution via centrifugation to obtain bacterial slurry, which served as the test solution for the 0% moisture content group. For other moisture content groups, bacterial solution was mixed uniformly with expanded perlite according to the specified ratio and placed in sterilized and dried beakers. The beakers were sealed with plastic wrap and stored indoors.

When preparing the sample, sterilize and dry the beaker, then weigh its mass M_1_. Remove the beaker that has reached the test age, pour in 81.73 g of nutrient solution, seal it with cling film, and place it in a 30°C constant temperature oven. After 3 days, pour the mass M_2_ onto the paper and place it on the tray. Then, place the beaker M_3_ and paper M_4_ in a 55°C oven until the mass M no longer changes, and calculate the amount of precipitation M_w_ (equation 2). In the equation, M is the quality of expanded perlite, and M_0_ is the initial quality of expanded perlite.


(2) $$M_{w}=M-M_{0}+M_{3}-M_{1}+M_{4}-M_{2}.$$

## RESULTS

### Changes in microbial concentration and urease activity

#### Changes in microbial spore concentration and urease activity affected by environmental pH

**Fig 1:**
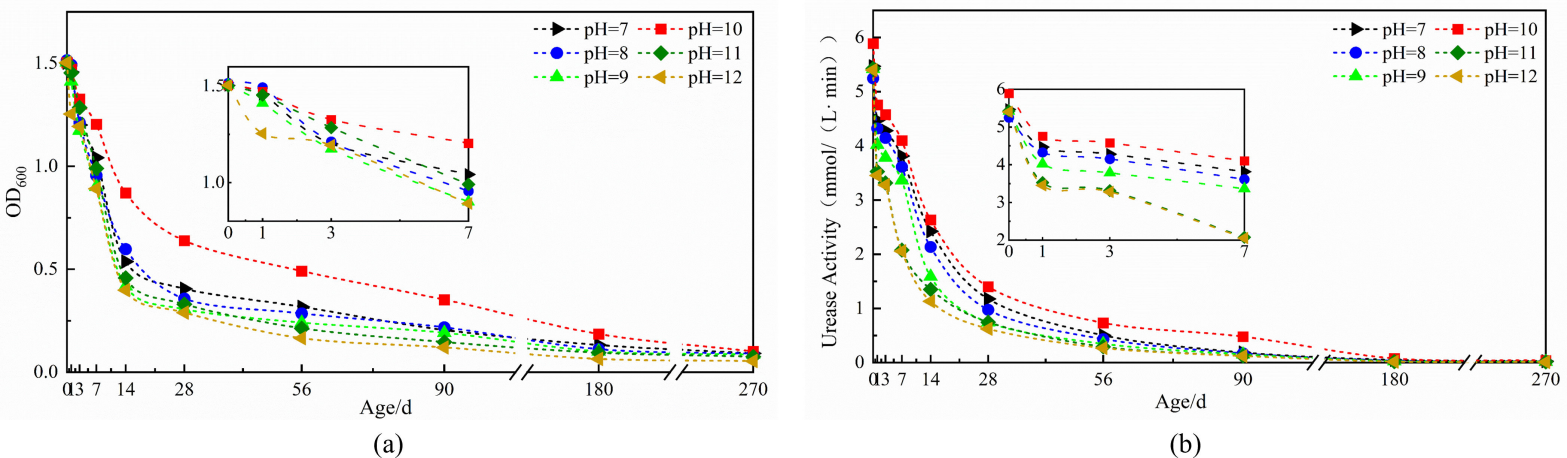
The curve of microbial spore concentration and urease activity affected by pH: (**a**) spore concentration, (**b**) urease activity.

The curves (Fig. 1) demonstrate that urease activity in *Bacillus pasteurii* spores exhibits a pH-dependent decline pattern consistent with the reduction in spore concentration over time. This correlation aligns with the findings reported by Su et al., showing a progressive reduction in both parameters across various pH conditions as incubation duration increases (47).

The pH = 10 simulated cement solution maintained higher spore viability and urease activity compared to other pH conditions at equivalent time points (Fig. 1). During the initial 0–3 day period, spore survival rates ranged from 78.01% to 88.26%, while urease activity remained between 5.25 and 5.89 mmol/(L·min). Both parameters showed minimal variation across different pH values during this phase, indicating that high spore concentrations confer relative pH insensitivity to urease activity. However, pH sensitivity increased progressively with spore aging (Fig. 1). After 14 days, the pH = 10 solution sustained a spore concentration of 0.87 (57.95% survival), significantly outperforming both lower pH solutions (average concentration: 0.517, 34.31% survival) and higher pH solutions (average concentration: 0.429, 28.51% survival) (Fig. 1a). This pH-dependent viability advantage persisted through longer incubation periods, with pH = 10 demonstrating 23.42% and 12.31% survival rates at 90 and 180 days, respectively. These values exceeded average survival in pH <10 solutions by 9.87% and 4.5%, and surpassed pH >10 solutions by 14.56% and 7.0% at the respective time points (Fig. 1a).

In simulated solutions at pH = 10, spore urease activity decreased by only 22.26% after 3 days, compared to average reductions of 24.28% in pH <10 solutions and 39.10% in pH >10 solutions (Fig. 1b). By day 56, urease activity in pH = 10 solutions maintained 0.73 mmol/(L·min), significantly higher than the averages of 0.43 mmol/(L·min) in lower pH environments and 0.28 mmol/(L·min) in higher pH conditions. While prolonged alkaline exposure substantially reduced urease activity across all groups, spores in pH = 10 solutions retained 0.49 mmol/(L·min) activity at 90 days—a marked contrast to the negligible activity observed in other pH conditions (Fig. 1b). These results demonstrate considerable alkaline tolerance in microbial spores, though high-alkaline environments (pH > 10) progressively exert greater detrimental effects on spore viability than low-alkaline conditions (pH < 10), particularly during medium to long-term exposure. This differential tolerance is attributed to the structural robustness of spore cell walls, which provides enhanced resistance to alkaline corrosion.

#### Changes in microbial spore concentration and urease activity affected by temperature

**Fig 2:**
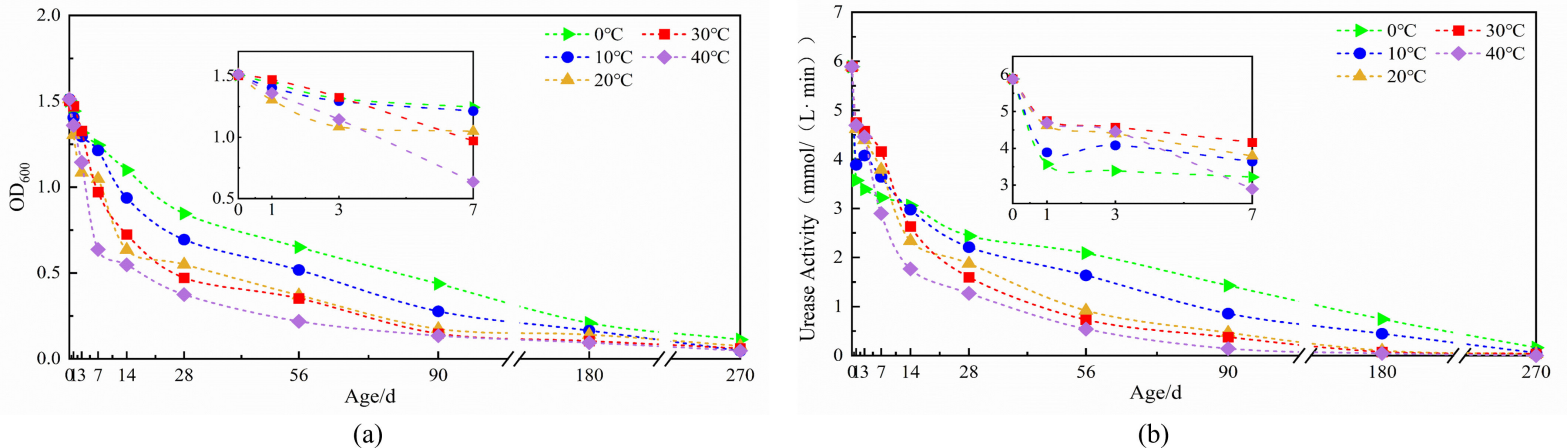
The curve of microbial spore concentration and urease activity affected by temperature: (**a**) spore concentration, (**b**) urease activity.

Temperature-dependent analysis reveals consistent declining trends in both urease activity and spore concentration across all temperature conditions during aging (Fig. 2). Initial measurements showed minimal divergence between 0°C and 30°C environments, with merely 1.90% difference in survival rates at day 1. During the initial 0–3 day period, optimal urease activity reached 4.76 mmol/(L·min) at 30°C (Fig. 2). However, temperature sensitivity increased substantially with prolonged exposure, evidenced by a 24.98% survival rate differential at 14 days. Both 20°C and 40°C conditions exhibited significant reductions in metabolic function and viability, with urease activity declining by 21.62% and 20.29%, and survival rates decreasing by 11.68% and 8.02%, respectively (Fig. 2). While early-stage spores demonstrated peak performance at 30°C with 97.87% initial survival, prolonged exposure to 20°C–40°C environments resulted in rapid decreases of both parameters. In contrast, 0°C–10°C conditions maintained relatively stable urease activity with minimal reduction (Fig. 2). Although both heating and cooling decreased urease activity and spore viability, heating had a more inhibitory impact because higher temperatures inactivate spore cell structures. After 180 days, the 0°C environment preserved substantially higher functionality (12.31% survival, 0.74 mmol/[L·min] urease activity) compared to 30°C conditions (6.14% survival, 0.08 mmol/[L·min]) (Fig. 2), establishing that lower temperatures better maintain long-term spore viability and enzymatic activity.

### Analysis of microbial mineral precipitation results

#### Changes in mineral precipitation by microbial spores under fixed and unfixed conditions affected by environmental pH

**Fig 3:**
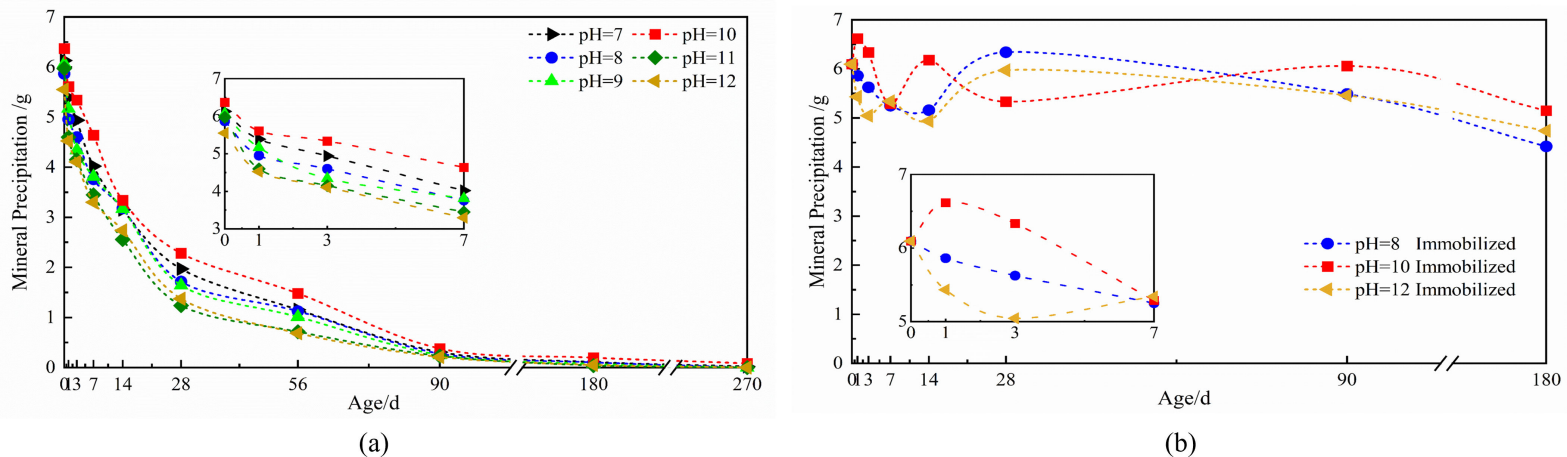
The curve of mineral precipitation of immobilized and non-immobilized *Bacillus pasteurii* spores affected by pH: (**a**) non-immobilized, (**b**) immobilized.

The relationship between pH and microbial-induced mineral precipitation reveals a consistent decline in precipitation across all pH levels as aging progresses (Fig. 3a), aligning with the observed patterns in spore concentration and urease activity. Throughout the experimental period, the simulated solution at pH = 10 yielded the highest mineral precipitation, with an initial precipitation value of 6.37 g (Fig. 3a). This superior performance demonstrates the critical role of optimal pH conditions in maintaining microbial mineralization capacity over extended durations.

**Fig 4:**
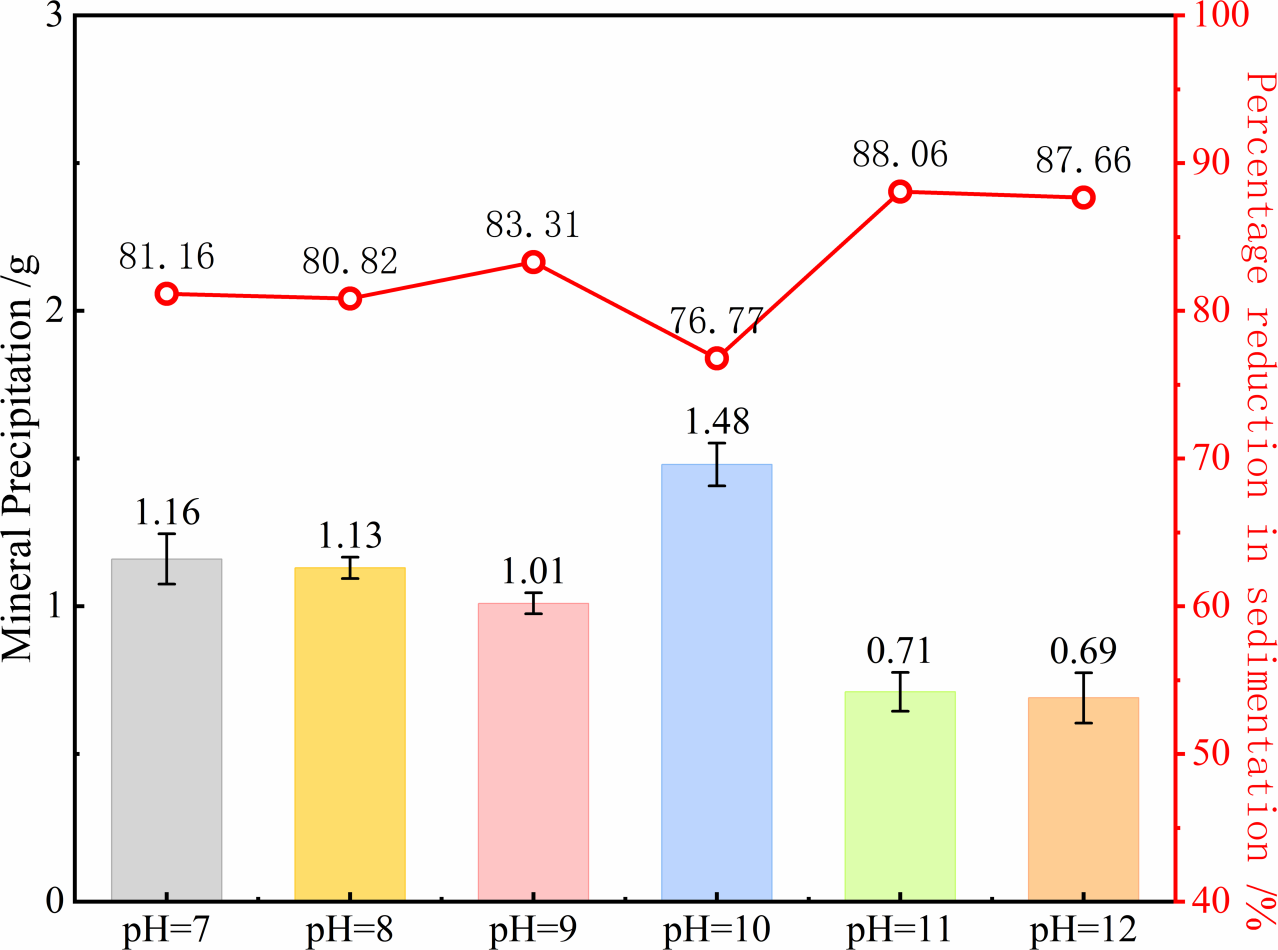
The amount and percentage of mineral precipitation of microbial spores at 56 days.

The mineral precipitation and percentage reduction at 56 days for microbial spores (Fig. 4), the impact of ambient pH on mineral precipitation progressively rises with age. Spore-induced mineral precipitation in a pH = 10 simulated solution at 56 days was 1.48 g, which was 76.77% less than the initial precipitation. The precipitation dropped by 81.76% in solutions with a pH of less than 10 and by 87.86% in solutions with a pH of greater than 10 (Fig. 4). These findings establish pH = 10 as the optimal alkaline environment for sustaining long-term microbial mineralization capacity, with progressively greater inhibitory effects observed under both higher and lower pH conditions as aging progresses.

The curve depicting the change in mineral precipitation caused by immobilized microbial spores under the influence of pH is displayed (Fig. 3b). The data demonstrate that immobilized spores maintain consistent mineral precipitation, averaging approximately 5.60 g, with minimal variation across different pH levels and aging periods. Maximum precipitation occurred at pH = 10, achieving an average of 5.88 g throughout the testing duration—surpassing low-alkaline environments by 0.35 g and high-alkaline conditions by 0.50 g (Fig. 3b).

**Fig 5:**
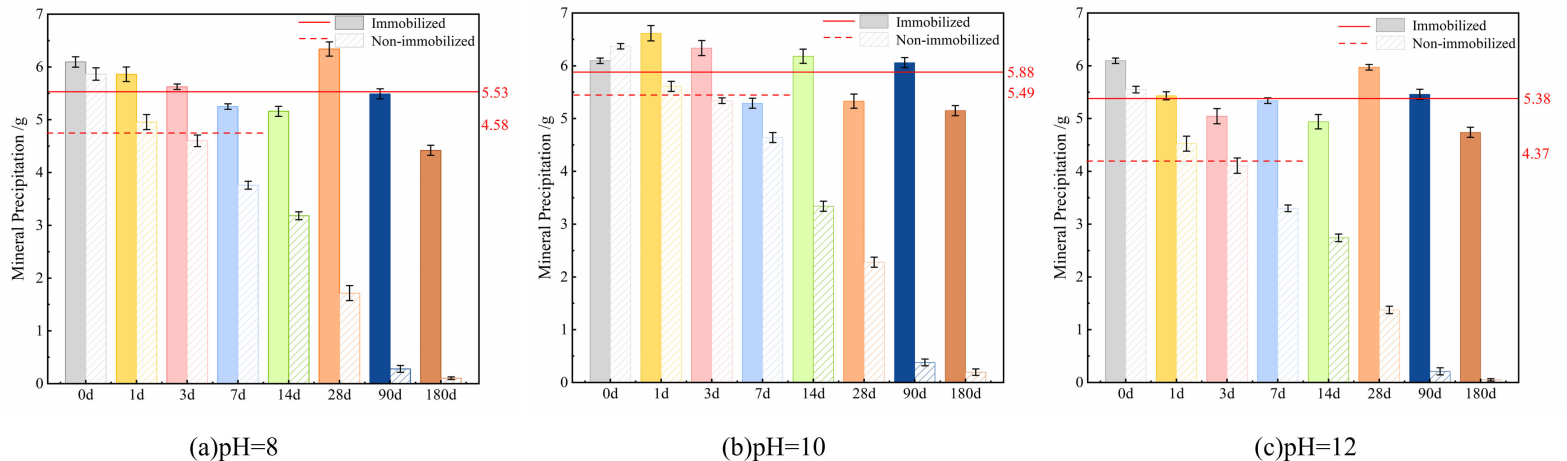
Comparison of mineral precipitation induced by immobilized and non-immobilized spores in pH 8 (**a**), 10 (**b**), and 12 (**c**) solutions.

Comparative analysis between immobilized and non-immobilized systems (Fig. 5) reveals distinct temporal patterns. Non-immobilized spores initially showed increased precipitation during 0–7 days, with averages of 4.8 g (pH = 8), 5.49 g (pH = 10), and 4.37 g (pH = 12), yet exhibited rapid decline thereafter, falling below 0.5 g by 90 days. In contrast, immobilized spores maintained stable precipitation throughout aging, sustaining 4.77 g at 180 days. The pH = 10 environment supported optimal spore adaptation and mineralization efficiency, while immobilized spores demonstrated remarkably consistent performance with only 0.39 g variation compared to non-immobilized counterparts (Fig. 5). These results confirm that expanded perlite immobilization effectively mitigates alkaline corrosion, enabling sustained microbial mineralization capacity over extended periods. The carrier system preserves spore functionality against pH-induced impact, maintaining high mineralization potential despite prolonged exposure to alkaline conditions.

#### Changes in mineral precipitation amounts of microbial spores under fixed and unfixed conditions, affected by temperature

**Fig 6:**
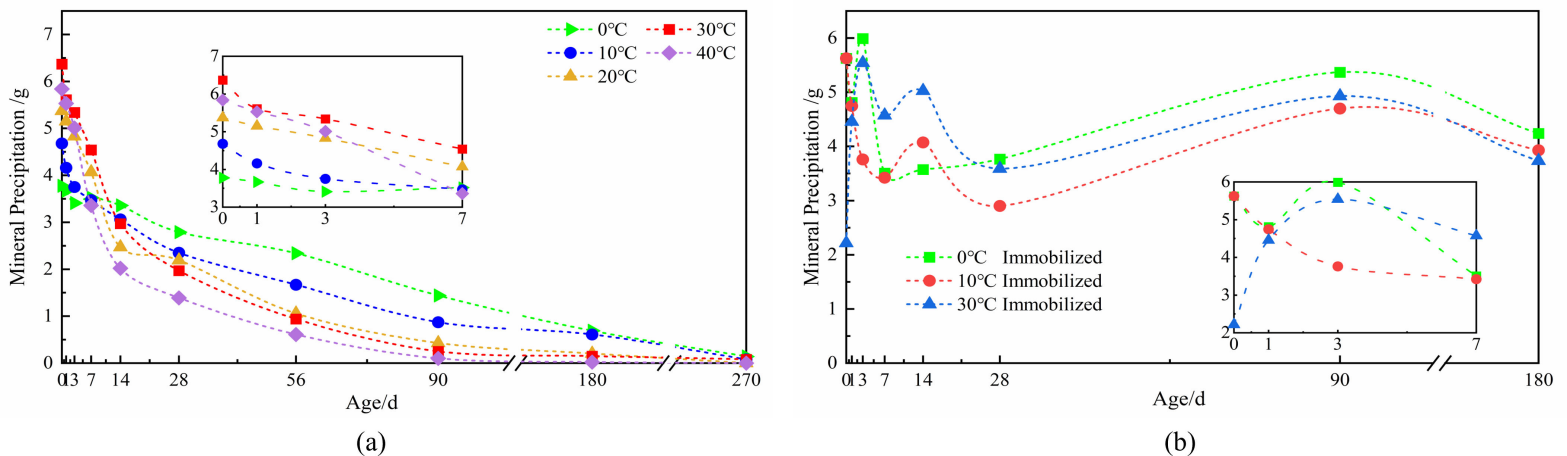
The curve of mineral precipitation of immobilized and non-immobilized *Bacillus pasteurii* spores affected by temperature: (**a**) non-immobilized, (**b**) immobilized.

The kinetic rate of chemical processes and the equilibrium constant of precipitation reactions are both impacted by temperature (48). The curve depicting the change in mineral precipitation caused by *Bacillus pasteurii* spores with temperature (Fig. 6a) shows that 30°C is optimal for maximum mineral precipitation yield, achieving 6.37 g initially. However, mineral precipitation demonstrates substantial age-dependent reduction across the 10°C–40°C range. While the 10°C condition maintained 0.87 g precipitation at 90 days, the 40°C group diminished to merely 0.1 g, indicating enhanced long-term mineralization capacity at lower temperatures (Fig. 6a). Notably, the 0°C condition sustained stable precipitation during the initial 0–7 days before undergoing a linear decline, ultimately reaching 1.44 g at 90 days—representing a 1.66-fold advantage over the 10°C group and confirming superior long-term mineralization performance at 0°C (Fig. 6a).

The aforementioned illustrates how bacteria perform better over the long term at temperatures of 0°C, 10°C, and 30°C. To further investigate temperature effects on sustained mineralization, microorganism-immobilized expanded perlite carriers were maintained in a pH = 10 simulated solution. Mineralization was monitored over 270 days to assess thermal stability (Fig. 6b). The temperature-dependent mineralization profile of immobilized microorganisms reveals significantly reduced thermal sensitivity, with precipitation levels remaining stable across the tested range. Maximum precipitation averaged 4.61 g at 0°C, moderately exceeding values recorded at 10°C and 30°C (Fig. 6b). Throughout the 0–180 day period, mineral precipitation variation remained within 4.34 g across the 0°C–30°C temperature spectrum. After 180 days, the lowest recorded precipitation was 3.74 g, demonstrating minimal deviation despite extended immersion (Fig. 6b). These results indicate that the mineralization function of immobilized microorganisms remains remarkably stable, exhibiting negligible changes in both temperature fluctuations and aging.

**Fig 7:**
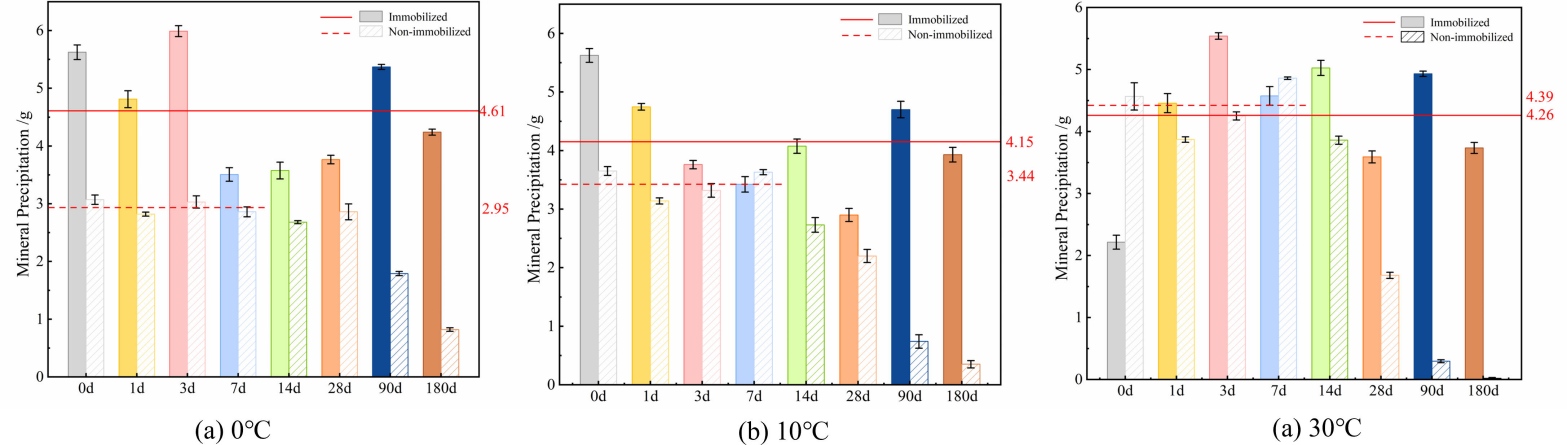
Comparison of the amount of mineral precipitation induced by immobilized and non-immobilized microorganisms at 0°C (**a**), 10°C (**b**), and 30°C (**c**).

The curve compares temperature-dependent mineral precipitation between immobilized and non-immobilized microorganisms (Fig. 7). Non-immobilized systems exhibited significant thermal sensitivity, with average precipitation values of 2.95 g (0°C), 3.44 g (10°C), and 4.39 g (30°C) during the initial 0–7 days. This pronounced temperature dependence confirms that microbial mineralization capacity in non-immobilized states is strongly influenced by thermal conditions, with optimal short-term performance at 30°C but rapid decline thereafter (Fig. 7). In contrast, immobilized microorganisms demonstrated remarkably stable precipitation across temperatures, with minimal variation throughout the testing period. The expanded perlite carrier effectively buffered thermal fluctuations, maintaining consistent microbial functionality. Notably, while immobilization enhanced precipitation at suboptimal temperatures (0°C–10°C), it moderately reduced output at the optimal growth temperature (30°C), indicating a protective trade-off that prioritizes long-term stability over peak short-term performance (Fig. 7). These results establish that carrier-based immobilization transforms microbial temperature response patterns, extending functional longevity particularly under non-ideal thermal conditions. The technology demonstrates particular value for applications where temperature stability outweighs the need for maximum initial mineralization rates (Fig. 7).

### Analysis of the results of the effect of moisture content on the amount of microbial mineral precipitation

To address the progressive moisture reduction during cement hydration that creates a desiccated internal environment, we established two experimental groups with distinct moisture regimes. The high-moisture group (60%–80% moisture content) was monitored for mineral precipitation variations during the initial 0–56 day period. Simultaneously, the low-moisture group (0%–40% moisture content) was evaluated over an extended duration (0–270 days) to assess long-term induced mineralization under moisture-deficient conditions that simulate aged concrete environments.

**Fig 8:**
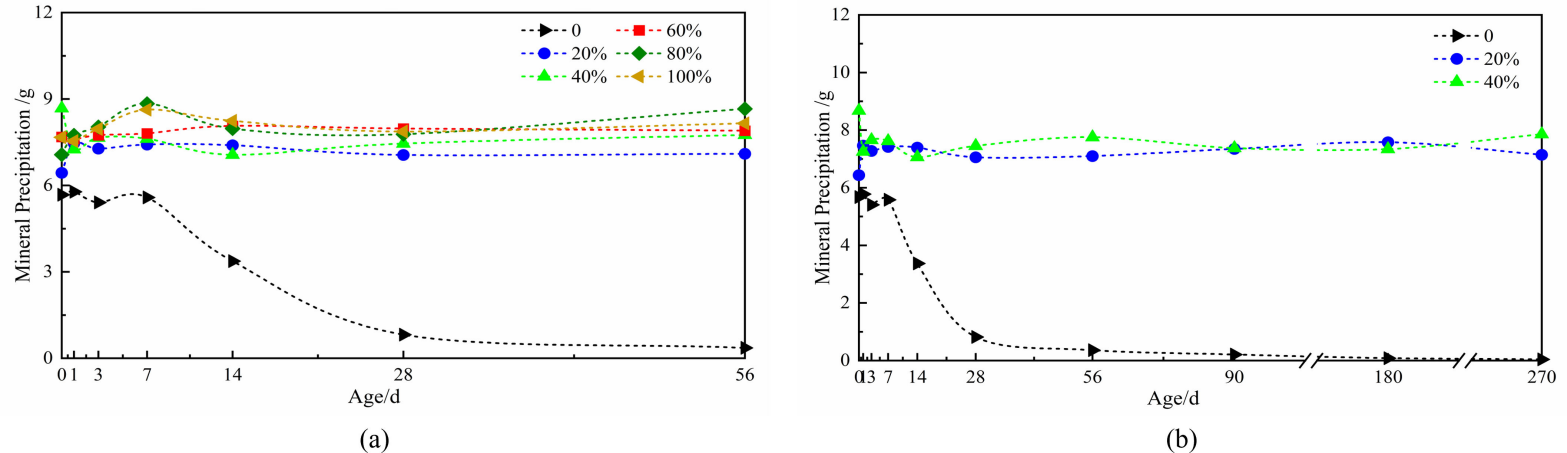
The curve of mineral deposition induced by *Bacillus pasteurii* spores affected by moisture content: (**a**) the moisture content of 0–56 days, (**b**) low moisture content of 0–270 days.

The curve illustrates moisture-dependent variations in spore-induced mineral precipitation (Fig. 8a). The high moisture group (60%–80%) demonstrated marginally greater precipitation (average difference: 0.54 g) than the low moisture group during the initial 0–56 day period. However, across the 20%–100% moisture range, mineral precipitation remained remarkably stable at approximately 7.91 g (Fig. 8a). Long-term analysis under low moisture conditions (Fig. 8b) reveals distinct behavior patterns. At 0% moisture, precipitation remained initially stable but underwent a rapid linear decline after 7 days, falling below 1 g by day 28. In contrast, the 20% and 40% moisture groups maintained stable precipitation levels of 7.29 g and 7.58 g, respectively, throughout the testing period. These results demonstrate that while complete desiccation severely compromises mineralization capacity, moisture levels ≥20% sufficiently support sustained microbial function (Fig. 8b). The expanded perlite carrier effectively maintains microbial viability and mineralization capability independent of moisture fluctuations, confirming its crucial role in preserving long-term microbial functionality in solid-loaded systems.

## DISCUSSION

### pH and temperature

We added dormant spores of microorganisms to a solution of simulated cementitious pores. Throughout the aging process, progressive spore activation triggered urea hydrolysis and subsequent calcium carbonate precipitation. During the initial 0–3 day non-carrier survival phase, environmental pH and temperature demonstrated no significant impact on microbial concentration, urease activity, or mineralization function. However, after 7 days, these parameters became substantially influenced by both factors, with lower temperatures proving more favorable for long-term microbial persistence. The internal concrete microenvironment typically maintains pH 11–13 due to hydration reactions, though crack formation and water infiltration reduce this to approximately pH = 10 at the fracture site (49). This study utilized domesticated spores featuring thickened cell walls and enhanced stress tolerance, enabling improved adaptation to concrete crack conditions and more effective mineralization capability. While initial testing showed high bacterial activity and concentration due to early resistance to alkaline erosion, extended immersion resulted in hydrolytic degradation of the spore coat, diminishing protection of the core (47, 50). Consequently, the 180-day spore survival rate reached only 12.31% at pH = 10. The urease activity and the mineralization capacity of bacteria show similar patterns of change with spore activity, according to the examination of the aforementioned experimental data. Additionally, a major element affecting their ability to mineralize is spore urease activity. Enzymatic reactions are the main component of the mineralization principle of urease-producing bacteria. Raising the temperature accelerates the substrate’s rate of dissolution by improving the substrate’s and air’s expansion coefficients (51). The temperature range between 30°C and 40°C is when microbial urease activity is highest (52). However, prolonged incubation at elevated temperatures induced thermal inactivation, causing rapid activity decline (51). After 180 days, urease activity maintained 0.74 mmol/(L·min) at 0°C, but diminished to 0.08 mmol/(L·min) at 30°C, demonstrating that lower temperatures better preserve long-term microbial urease activity and mineralization efficacy.

**Fig 9:**
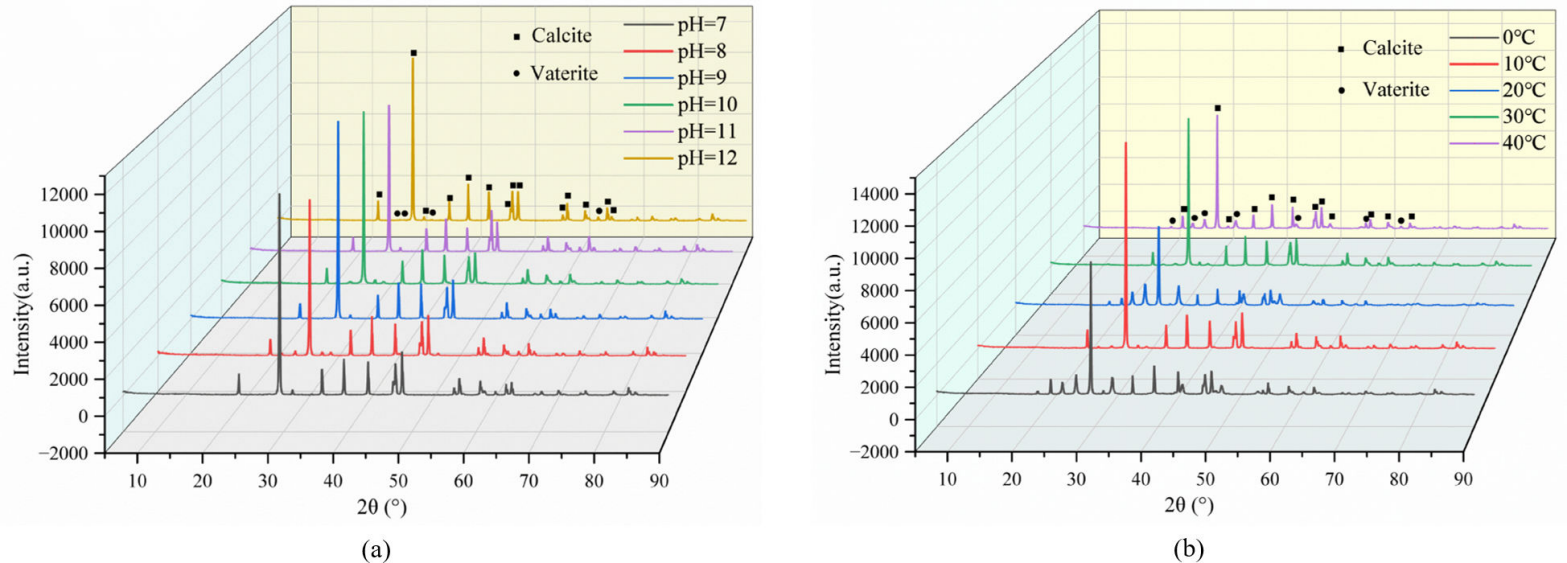
XRD phase analysis of mineral precipitation of microorganisms in all pH and temperature environments: (**a**) pH, (**b**) temperature.

The crystal structure and microstructure of spore-induced mineral precipitates under varying environmental conditions were characterized using X-ray diffraction (XRD) and scanning electron microscopy (SEM). XRD analysis revealed distinct diffraction patterns for microbially induced minerals, with characteristic peaks at approximately 2θ = 63° for vaterite and 2θ = 29.4° for calcite (Fig. 9). The precipitates consisted primarily of calcite and vaterite polymorphs. While the positions of characteristic diffraction peaks remained consistent across different pH conditions with only minor intensity variations, temperature significantly influenced crystal phase composition (Fig. 9). The maximum calcite content was observed at 30°C, with higher temperatures promoting vaterite formation at the expense of calcite. Throughout all conditions, the calcite diffraction peaks demonstrated markedly greater intensity than vaterite peaks, establishing calcite as the dominant crystalline phase in microbially induced calcium carbonate precipitation (Fig. 9).

**Fig 10:**
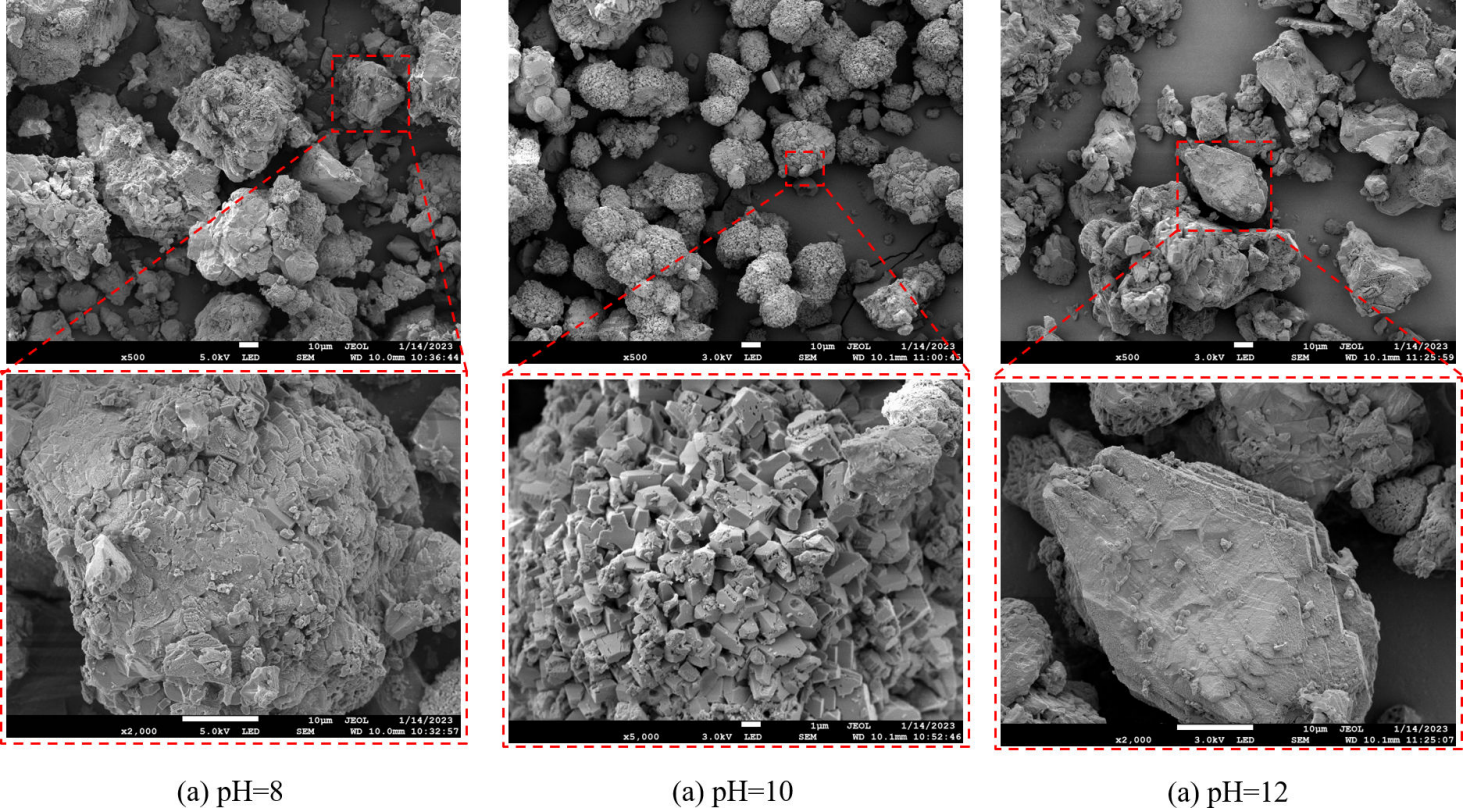
SEM micro-morphology of microbially induced mineral precipitation at pH (**a**) 8, (**b**) 10, and (**c**) 12.

**Fig 11:**
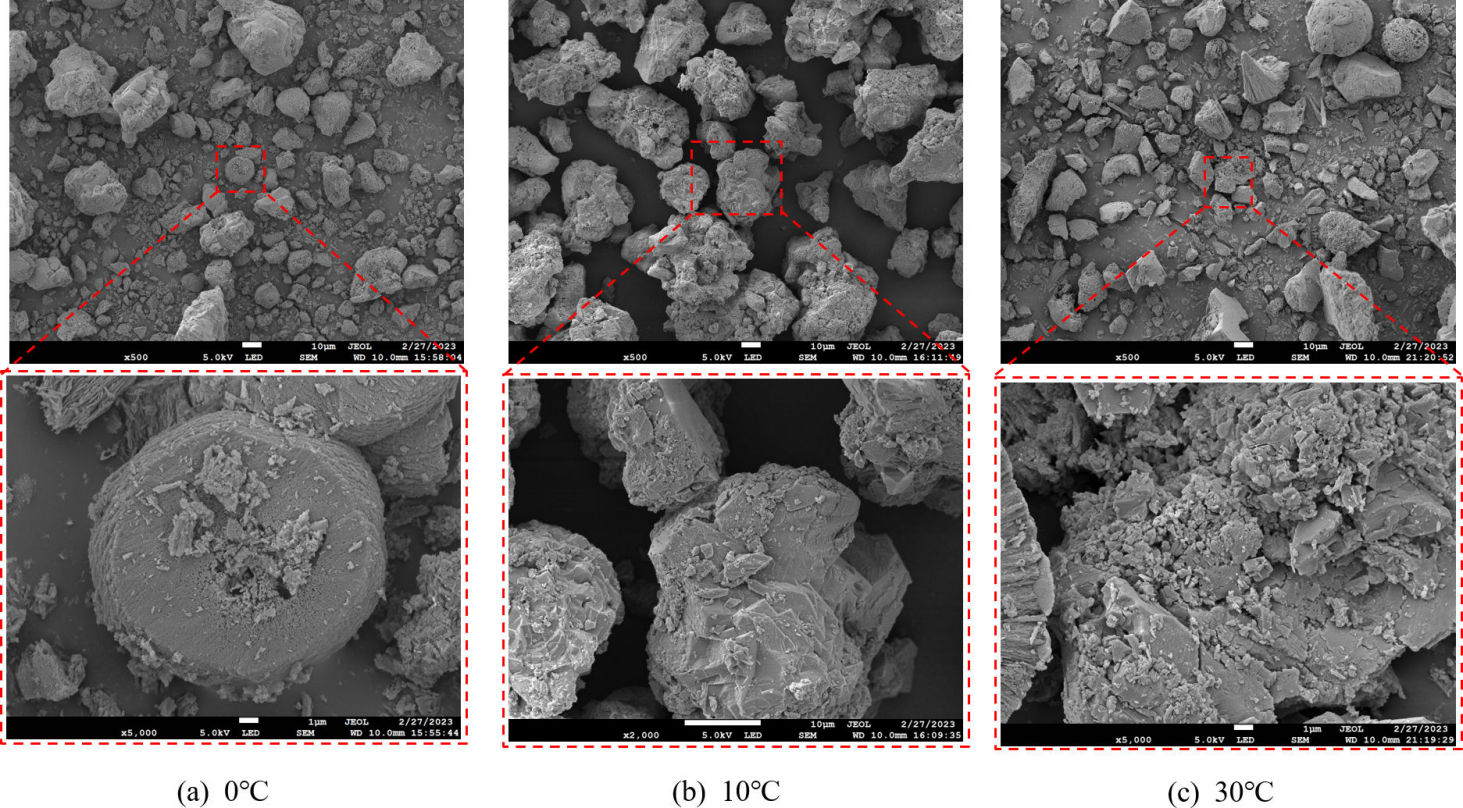
SEM micro-morphology of microbially induced mineral precipitation at (**a**) 0°C, (**b**) 10°C, and (**c**) 30°C.

The SEM analysis revealed distinct microstructural variations in calcium carbonate precipitates formed under different environmental conditions (Fig. 10 and 11). In both neutral and strongly alkaline environments, microbially induced calcium carbonate particles exhibited irregular morphologies with textured surfaces. At pH = 10, particles assembled into composite spherical aggregates (13.79–21.93 μm) through the stacking of hexagonal prismatic subunits, demonstrating well-defined crystalline features, effective filling capacity, and structural stability (Fig. 10). Temperature significantly influenced crystal morphology, with increasing temperature correlating with enhanced surface roughness and larger particle sizes. At 30°C, particles formed spherical structures (0.52–1.72 μm) composed of stacked hexagonal prisms (Fig. 11), which exhibited strong adhesion properties but limited suitability for sustained bacterial mineralization.

### Expanded perlite solid load

Expanded perlite (EP) serves as an economical and widely adopted concrete additive due to its stable physicochemical properties. Its highly porous structure provides substantial adsorption capacity and abundant attachment sites for microorganisms. When employed as a microbial carrier, EP effectively shields bacteria from the highly alkaline concrete environment, thereby sustaining long-term mineralization capability. Microbial mineralization precipitation reached 5.6 g when using EP immobilization—a 28-fold increase compared to non-immobilized conditions at 90 days. This research establishes that microbial immobilization enhances urease activity, consequently increasing mineral precipitation (53). Furthermore, the high porosity and water absorption capacity of EP maintain stable internal moisture levels, providing the aqueous environment necessary for spore activation and biomineralization. However, this same porosity may absorb mixing water during concrete processing, potentially leading to microbial detachment and reduced hydration efficiency. At higher dosage levels, these effects become more pronounced, significantly diminishing both the mechanical properties of the concrete matrix and the interfacial performance between the matrix and expanded perlite particles. To address these limitations, this study proposes a “porous material + sugar coating layer + protective layer” structure, where EP particles are encapsulated within a high-strength hydraulic material. This design demonstrates excellent compatibility with conventional concrete mixing procedures. Incorporating EP particles (1.18–2.36 mm diameter) during initial mixing effectively refines the concrete pore structure, reduces macro-voids, and mitigates stress concentration from particle impact (41, 54, 55). Additionally, this configuration prevents premature nutrient-microbe contact that could trigger germination, while avoiding simultaneous immobilization that would reduce repair capacity (42, 45, 56). The sugar-coated EP microbial carrier maintains effective crack repair after 180 days, demonstrating that sustained cellular activity and mineralization significantly enhance both compressive strength and self-healing performance (21).

This investigation focuses on individual parameters (pH, temperature, moisture) affecting mineralization patterns, precipitate morphology, and microbial longevity. However, it does not address the synergistic effects of multiple factors. Future research should examine microbial long-term activity under combined environmental influences, as induced mineralization and remediation performance result from complex interactions among multiple variables.

### Conclusion

1. Temperature and pH significantly influence the mineralization capacity and long-term survival characteristics of spores in non-immobilized conditions. Both urease activity and spore concentration exhibit a declining trend during aging. Microbial spores demonstrate superior long-term viability and enhanced mineralization capability at 0°C and pH = 10 compared to other environmental conditions. High-alkaline environments cause substantially greater microbial corrosion than neutral or moderately alkaline conditions. Although elevated temperatures accelerate metabolic processes, lower temperatures prove more conducive to extended microbial survival, with microbial activity being more adversely affected by thermal stress than by cold exposure.
2. In immobilized systems, temperature and pH exert minimal influence on long-term microbial mineralization function. Mineral precipitation remains largely consistent, stabilizing at approximately 5.60 g. After 90 days at 0°C and pH = 10, the mineralization rate under immobilized conditions demonstrates a 28-fold enhancement compared to non-immobilized systems. Expanded perlite effectively mitigates the detrimental long-term effects of high alkalinity and temperature fluctuations on microorganisms, thereby extending their functional lifespan and significantly improving sustained induced mineralization capacity.
3. Microbial-induced mineral precipitation remained stable in the 0% moisture group during the initial 0–7 days, though at lower levels than observed in moist conditions. Beyond this period, mineralization capacity declined rapidly. Moisture levels between 20% and 100% demonstrated no significant difference in long-term microbial mineralization function, though slightly higher precipitation occurred in high-moisture conditions. These findings confirm that while an aqueous environment is essential for microbial mineralization, the process remains largely unaffected by moisture content variations within this range. Furthermore, employing expanded perlite as a carrier effectively maintains internal moisture, thereby supporting sustained microbial mineralization capacity over extended durations.
